# Construction and application of sustained-release active intelligent packaging incorporating phenolic acid copigmented *Aronia melanocarpa* anthocyanins and microencapsulated carvacrol

**DOI:** 10.1016/j.fochx.2025.103384

**Published:** 2025-12-13

**Authors:** Liu Yang, Xue Ding, Rui Xiao, Xinyu Zhang, Zhipeng Jiang, Wenwen Chen, Shengyu Xu, Hongyuan Zhang

**Affiliations:** a College of Food Science and Engineering, Changchun University, Changchun 130022, China; b Key Laboratory for Clean Energy in Western Jilin, Chemistry College, Baicheng Normal University, Baicheng 137000, China; c Baishan City Lin Yuan Chun Ecological Science and Technology Co, Baishan 134300, China

**Keywords:** Active intelligent film, Copigmentation, Anthocyanin, Bacteriostasis, Color indication, Fresh shrimp preservation

## Abstract

Active intelligent packaging often suffers from insufficient indicator sensitivity and limited antibacterial duration. To overcome these limitations, the system incorporates *Aronia melanocarpa* anthocyanins (AMA) copigmented by phenolic acid, carvacrol (CL) encapsulated by hydroxypropyl-β-cyclodextrin (HP-β-CD), and integrates these components into potato starch (PS) to produce a multifunctional film with enhanced indicator sensitivity and sustained release properties. The results revealed that ferulic acid (FA) provided notable stabilizing effect on AMA, maintaining ΔE < 1 after two cycles of acid-base alternation. The incorporation of HP-β-CD allowed for efficient encapsulation of CL through hydrogen bonding, achieving sustained release over 96 h. The incorporation of FA-AMA and CL@HP-β-CD system improved the roughness, UV-blocking, and antioxidant performance of the film. Additionally, the film extended shrimp shelf life by 12 h, with a visible transition from pink to blue upon spoilage. This research offers a novel approach to the advancement of active intelligent packaging.

## Highlights

• FA copigmented AMA stabilizes AMA to enhance the color sensitivity of films.
• CL was embedded to achieve slow-release antibacterial.
• Construct a packaging with both freshness indication and long-term antibacterial.
• The film extends shrimp shelf life by 12 h through non-contact preservation.

## 1 Introduction

Shrimp are appreciated by consumers for their taste and nutritional value (Kim et al., 2020). However, their high susceptibility to rapid spoilage stems from elevated endogenous enzyme activity, coupled with a high moisture content and substantial protein content (Wu et al., 2021). This rapid spoilage leads to considerable economic losses within the shrimp supply chain, while also presenting potential health hazards to consumers (Peng et al., 2022). Recent years have seen the introduction of several innovative technologies aimed at preserving shrimp. Liu et al. (2023) applied high-voltage electrostatic fields to freeze shrimp, minimizing muscle tissue damage and delaying spoilage. Ji et al. (2024) developed Pickering emulsions using modified cellulose nanocrystals and cinnamaldehyde, which effectively inhibited the psychrophilic bacteria responsible for shrimp spoilage under refrigeration. Yang, Zhuo, et al. (2024) created gelatin-oxidized ricin cryogels as reusable ice cubes, greatly enhancing the preservation of shrimp. However, these techniques focus only on preservation and do not allow for the real-time monitoring of shrimp freshness. This has led to the rise of active intelligent packaging as a key research area. Kim et al. (2022) introduced an intelligent packaging film made from gelatin, agar, butterfly pea flower anthocyanins, and zinc oxide, which not only preserved shrimp but also monitored freshness during cold storage. Wang et al. (2022) incorporated alizarin as an indicator and lavender essential oil as an antibacterial agent into a gelatin film, enabling both preservation and real-time freshness detection. Despite these advancements, limitations such as rapid antibacterial substance release, indicator instability, and direct contact with shrimp surfaces remain. Consequently, the development of dual-functional active intelligent packaging with both high-sensitivity indicators and long-lasting antibacterial properties has become a current research focus in shrimp preservation and freshness monitoring.

Essential oils, derived from plants, are volatile and hydrophobic natural compounds. They are recognized for their non-toxic properties and possess bioactivities, including antimicrobial and antioxidant effects, which fulfill the growing demand for food safety in both the food industry and consumer sectors. The integration of these oils into biodegradable films for creating active packaging solutions aimed at food preservation has gained significant research interest (Farooq et al., 2023). As natural additives, these plant-derived chemicals have captured attention for the development of active biodegradable films that cater to consumer preferences for chemical-free food preservation. CL, a phenolic monoterpenoid mainly present in aromatic plant oils, demonstrates broad-spectrum antimicrobial activity along with insecticidal, antimutagenic, and antioxidant effects (Zhi et al., 2025). Despite these properties, its practical application faces challenges due to its physicochemical instability and susceptibility to thermal, photolytic, and oxidative stress (Lucas-González et al., 2023). When directly incorporated into hydrophilic film matrices, CL leads to phase separation, odor release, and a reduction in the structural integrity of the film, thereby compromising its bioactivity. To address these issues, encapsulation methods have been developed to stabilize volatile compounds and facilitate controlled release over time. HP-β-CD, with its amphiphilic cavity structure that supports host-guest interactions, offers a powerful means to thermodynamically stabilize essential oil components through molecular encapsulation (Dos Santos & Anconi, 2024). This system holds promise for achieving long-lasting antimicrobial action in dual-function films via slow-release mechanisms.

Anthocyanins are water-soluble polyphenolic substances with antioxidant effects (Dong et al., 2023). Their structural changes in response to varying pH levels lead to color shifts, making them ideal for real-time monitoring of food freshness. Study have incorporated roselle anthocyanins into film matrices to develop pH-indicating packaging with effective meat freshness monitoring capabilities (Huang et al., 2022). Ma et al. (2024) developed pH-responsive films from blueberry anthocyanins to monitor the freshness of pork. Compared to grapes, AMA content is approximately 80 times higher, and it exceeds blueberries by more than fivefold, which makes them widely used in functional foods due to their affordability and ease of access. However, anthocyanins can degrade under improper processing conditions, including excessive temperature and extended processing times, which diminishes both the color stability and the sensitivity of smart packaging systems (Zang et al., 2022). Improving the color stability of anthocyanins is crucial for the broader application of smart packaging technologies. To enhance this stability, methods like acylation, encapsulation, and copigmentation have been developed (Li et al., 2024). Among these, copigmentation is considered a more convenient strategy for stabilizing anthocyanin-based intelligent films. For instance, Chen et al. (2025) demonstrated the use of selenopeptides combined with grape skin anthocyanins to produce highly stable and multifunctional intelligent films. In another study, Huang et al. (2023) showed that adding oxalic acid to roselle anthocyanin/polyvinyl alcohol/hydroxymethyl cellulose films significantly improved their mechanical properties. Unlike metal ions or large biomacromolecules, phenolic acids are small molecules that not only improve the color intensity and brightness of anthocyanins but also pose no food safety risks (Zhang et al., 2021). Phenolic acids enhance color and stability primarily by reducing the structural conversion of anthocyanins to the colorless chalcone form (Chung et al., 2015). Despite their potential, research on using phenolic acids for copigmentation within film systems to improve anthocyanin color stability remains underexplored.

Given the environmental challenges posed by petroleum-based plastic films, there is a growing shift toward developing biodegradable smart films using natural biopolymers (Li et al., 2023). These biopolymers, such as polysaccharides, proteins, lipids, and biosynthesized polyesters, offer a more sustainable alternative (Jayakumar et al., 2022). These biodegradable materials effectively reduce the migration of aroma compounds and prevent the infiltration of harmful substances during storage, thus enhancing food safety and preservation. Among these, PS has become a widely recognized biopolymer due to its low cost, abundant availability, unique paste rheology, and the natural barrier properties of starch-based films (Liu, Chen, et al., 2024). Despite these advantages, the use of PS in its pure form is limited by its brittleness, low tensile strength, susceptibility to oxidation, and lack of antimicrobial properties. To address these issues, Li, Mao, et al. (2025) added thymol microcapsules to starch, a method that not only improved the mechanical properties of the films but also extended the shelf life of cherry tomatoes. Similarly, Yu et al. (2025) integrated bilberry anthocyanins into starch films to create packaging with real-time monitoring capabilities. This modification not only enhanced the mechanical properties and UV-blocking ability of the films but also contributed to the preservation of milk. However, reports on combining both modified CL and anthocyanins in starch-based films are still relatively scarce.

Although previous studies have explored active intelligent films with pH-indicating and antibacterial properties, these films still suffer from critical limitations, such as insufficient sensitivity of the indicator and excessively rapid release of active substances. In this study, the film utilizes PS as a substrate, FA-copigmented AMA as the indicator, with HP-β-CD encapsulated CL (CL@HP-β-CD, IC) as the antibacterial agent. It is the first to achieves the sustained-release bacteriostasis of active agents and high-sensitivity response of the indicator. The physicochemical characteristics of the film were assessed using techniques like fourier transform infrared (FTIR) spectroscopy, thermogravimetric analysis (TGA), mechanical property testing, antioxidant activity assays, antibacterial performance evaluation, pH-responsive reversibility analysis, and microstructural imaging. The film was then applied to non-contact preservation and freshness monitoring of fresh shrimp, offering the benefits of extended shelf life, reduced waste, and enhanced commercial value. This research offers a novel approach to the advancement of active intelligent packaging while supporting the comprehensive use of *Aronia melanocarpa* resources.

## 2 Materials and methods

### 2.1 Materials

Fresh shrimp (25.78 ± 1.62 g) of *Litopenaeus vannamei* were sourced from a local Eurasian supermarket (Changchun, China). The preparation of *Aronia melanocarpa* anthocyanins (AMA) followed the procedure outlined by (Chen et al., 2024). Materials such as potato starch (PS), caffeic acid (CA), ferulic acid (FA), p-hydroxybenzoic acid (PHDA), gallic acid (GA), hydroxypropyl-β-cyclodextrin (HP-β-CD), and carvacrol (99.0 % purity, CL) were obtained from Shanghai Aladdin Biochemical Science and Technology Co. Ltd., Shanghai, China.

### 2.2 Phenolic acid cochromic AMA

#### 2.2.1 UV–vis absorption spectroscopy

The method outlined by (Li et al., 2023) was modified in this study. FA, CA, GA, PHDA, and AMA were combined in a 1:10 M ratio. A buffer solution with a pH range of 2–8 was prepared using citric acid and disodium hydrogen phosphate. The pigment solution was then blended with the buffer in a 1:5 ratio. This mixture was photographed, and the UV absorption spectrum was measured using a Shimadzu UV-2600 spectrophotometer (Kyoto, Japan), scanning from 350 to 800 nm.

#### 2.2.2 Effect of different molar ratios of phenolic acid in color supplementation

AMA was dissolved in ethanol solution adjusted to pH 3. FA, CA, GA, and PHDA were separately dissolved in anhydrous ethanol, and the solutions were mixed in molar ratios of 1:0, 1:1, 1:5, 1:10, 1:20, and 1:40. The absorption spectra were recorded within the wavelength range of 350–800 nm.

### 2.3 Preparation and characterization of IC

#### 2.3.1 Preparation of IC

To prepare IC, 5 g of HP-β-CD was dissolved in 45 mL of water and heated to 55 °C. The mixture was stirred at 467.5 ×g for 20 min until fully dissolved. CL, in a 1:5 (*v/v*) ratio with anhydrous ethanol, was added dropwise into the HP-β-CD solution via syringe. The resulting mixtures, at ratios of 1:6, 1:8, 1:10, and 1:12, were stirred for 2 h at 45 °C, then cooled to room temperature and reheated for an additional 2 h. After refrigeration at 4 °C overnight, the precipitate was collected by vacuum filtration, washed with anhydrous ethanol, and dried in a convection oven at 50 °C for 36 h. The IC was then dried to constant weight at 110 °C.

#### 2.3.2 Scanning electron microscope (SEM)

Scanning electron microscopy (SU3800, Hitachi, Tokyo, Japan) was employed to examine the IC microstructure. Prior to analysis, dry samples underwent sputtering for 2 min using an ion sputter coater, followed by coating with a thin conductive layer of Au—Pt alloy.

#### 2.3.3 Embedding rate

The maximum absorbance wavelength was identified through a full-wavelength scan (200–800 nm) of CL at a concentration of 4 μg/mL, using a UV-1800 spectrophotometer (UV-1800, Shimadzu, Kyoto, Japan). Solutions of CL in ethanol with varying concentrations (10–60 μg/mL) were prepared. Absorbance values were recorded and used to generate a standard curve, which was fitted with a linear regression model: y = 0.0153×﹣0.0224, R^2^ = 0.9994.

To dissociate IC, it was dissolved in 15 mL of anhydrous ethanol and sonicated for 10 min, which released CL from the cyclodextrin cavity into the ethanol, forming a uniform dispersion. The solution was then subjected to centrifugation at 5550 ×*g* for 15 min. After centrifugation, the supernatant was carefully collected, appropriately diluted, and analyzed by UV–Vis spectroscopy at 277 nm. Absorbance was measured in triplicate to determine the average value. The CL concentration was calculated by applying the absorbance to the standard curve equation, and the embedding efficiency was then determined.

(1) $$\mathrm{Embedding rate}\left(\%\right)=\frac{C_{1}}{C_{0}}$$

where C_1_ is the mass of CL in IC, and C_0_ is the mass of CL before embedding.

#### 2.3.4 FTIR spectroscopy

Intermolecular interactions in IC were studied using FTIR spectroscopy (Nicolet IS5, Thermo Fisher Scientific), which recorded spectra in the range of 400 to 4000 cm^−1^ with a resolution of 4 cm^−1^.

#### 2.3.5 TGA

TGA of IC was carried out with a thermogravimetric analyzer (STA409PC, Netzsch, Germany). The sample was heated from 30 to 800 °C at a rate of 10 °C/min under a nitrogen flow of 20 mL/min.

#### 2.3.6 Antimicrobial properties

The antimicrobial activity was assessed using a method adapted from a previous study (Diao et al., 2022). *Staphylococcus aureus* (*S. aureus*, G^+^) and *Escherichia coli* (*E. coli*, G^−^) were chosen as the model organisms. A bacterial suspension (∼10^6^ CFU/mL) was prepared, and IC solutions (0.625–80 mg/mL) were prepared by performing a two-fold serial dilution in 96-well plates. After an incubation period of 18–24 h at 37 °C, the minimum inhibitory concentration (MIC) was determined. To evaluate the minimum bactericidal concentration (MBC), 10 μL of the bacterial suspension was mixed with molten agar and incubated at 37 °C for 24 h. Inhibition of bacterial growth was assessed by visual observation.

### 2.4 Preparation and characterization of active intelligent film

#### 2.4.1 Preparation of active intelligent film

AMA was dissolved in a buffer solution at pH 3. FA was initially dissolved in anhydrous ethanol, then combined with the AMA solution to prepare a film-forming solution containing 0.2 mg/mL AMA and 1.6 mg/mL FA. Concurrently, IC was dissolved in deionized water to reach final concentrations of 20 and 40 mg/mL in the film-forming system. A separate aqueous solution of 25 mg/mL PS was heated to 63 °C under continuous stirring (275.5 ×g) until full gelatinization occurred. Once gelatinized, the IC dispersion was added to the PS and stirred for 1 h. The FA-AMA mixture was then incorporated with stirring for 30 min, followed by the addition of glycerol (35 % *w/w*, dry polymer basis). The pH was adjusted to 3.5 using 1 M HCl while maintaining constant stirring. Twenty milliliters of the film-forming solution were transferred to a petri dish and dried at 40 °C for 10 h. The films obtained were labeled as AMA/PS (control), FA-AMA/PS (FA modified), IC-AMA/PS (20 mg/mL IC), IC-FA-AMA/PS I (20 mg/mL IC + FA), and IC-FA-AMA/PS II (40 mg/mL IC + FA).

#### 2.4.2 Morphology

Film samples were placed onto aluminum stubs and coated with a 5 nm layer of Au—Pd under vacuum. The microstructure was examined using scanning electron microscopy (SU3800, Hitachi, Tokyo, Japan) at an accelerating voltage of 5 kV. Nanoscale morphology was analyzed via atomic force microscopy (AFM) (SPM9700, Shimadzu, Kyoto, Japan). The scanning was conducted at a 1 Hz rate in contact mode over a 2 × 2 μm^2^ area. Surface roughness (Ra, Rq) was determined using *Nanoscope* v1.5 software.

#### 2.4.3 Water vapor permeability (WVP)

WVP was determined following the method previously described (Hashim et al., 2023). The film specimens were positioned over beakers with 10 mL of deionized water in desiccators set to 25 % relative humidity and 25 °C. The mass of the beakers was measured daily for 5 d(day). Water adsorption capacity was calculated using the following formula:

(2) $$\mathrm{WVP}=\frac{\left(\Delta w\times L\right)}{\left(\mathrm{\Delta p}\times \mathrm{Sxt}\right)}$$

where WVP is water vapor permeability (g·m^−2^·d^−1^), Δw indicates the mass change of the conditioned film specimens (g), L represents the thickness of the film (mm), S is the area of the film (mm^2^), t is the time, and Δp is the vapor pressure difference calculated using the Antoine eq. (1.584 kPa at 25 °C).

#### 2.4.4 Mechanical properties

Tensile testing was performed in accordance with GB/T 1040.3–2006 using an electromechanical testing system (QJ210–50 N, Hua ye Science and Education Equipment Co., Ltd., Shanghai, China) (China & o. P. o. S. A. o. t. P. s. R. o., 2006). Rectangular specimens measuring 70 × 25 mm were cut using surgical steel blades. A dual-clamping system with precision-aligned grips was employed, maintaining a 40 mm initial span and a crosshead speed of 0.667 mm/s until fracture occurred. Stress-strain data were recorded at a sampling frequency of 1000 Hz. The tensile strength (TS) and elongation at break (EB) were calculated using the following equations:

(3) $$\mathrm{TS}\left(\mathrm{Mpa}\right)=F/S\#$$

(4) $$\begin{array}{c} \mathrm{EB}\left(\%\right)=\Delta L/L\times 100\# \end{array}$$

where F is the maximum force at the breaking point (N), S represents the initial cross-sectional area of the specimen (m^2^), ΔL is the displacement at fracture measured by the laser extensometer, and L is the initial distance between the grips (mm).

#### 2.4.5 Water contact angle (WCA)

The approach outlined by Yang, Wang, et al. (2024) was modified for this study. The WCA of the composite films was evaluated using the sessile drop technique. A 2 μL drop of distilled water was placed on the film surface, and digital images of the droplet were captured at designated time intervals. WCA values were then measured and computed using SCA20 software. Each composite film was tested in five replicates, with measurements taken at five randomly selected locations on the film surface.

#### 2.4.6 FTIR spectroscopy

FTIR spectroscopy (Nicolet IS5, Thermo Fisher Scientific) analyzed intermolecular interactions within film across 400–4000 cm^−1^ at 4 cm^−1^ resolution.

#### 2.4.7 Thermogravimetric analysis

The thermogravimetric stability of film was assessed using a thermogravimetric analyzer (STA409PC, Netzsch, Germany), maintaining a nitrogen flow rate of 20 mL/min and heating the sample from 30 to 800 °C at a thermal rate of 10 °C/min.

#### 2.4.8 X-ray diffraction (XRD)

The structural changes of the film were analyzed using an X-ray diffractometer (D8 ADVANCE A25, Bruker, Germany) with a scanning range of 5 to 90^°^ at 40 kV and 40 mA.

#### 2.4.9 Storage color stability

The films were stored at 4 and 25 °C for 30 d. Photographs of the films were captured using a digital camera, and chromaticity changes were recorded with a chroma meter at 5 d intervals. The initial color value of the film was used as the reference, and the calculations of Δ*E* were performed using the following formula:

(5) $$\begin{array}{c} \Delta E=\sqrt{{\left(L-L^{*}\right)}^{2}+{\left(a-a^{*}\right)}^{2}+{\left(b-b^{*}\right)}^{2}}\# \end{array}$$

where L*, a* and b* are the color parameters of the film. *L*, *a* and *b* are the initial color parameters of the film.

#### 2.4.10 pH response

Rectangular samples (10 mm × 10 mm) of the film were submerged in buffer solutions with pH values between 2 and 8 for 1 min. Digital images were taken, and color changes were quantified using a chroma meter (Chroma Meter CR-400, Konica Minolta, Japan). The initial color of the film served as the baseline, and ΔE was computed using the formula in Eq. (5).

#### 2.4.11 Copigmentation facilitated pH sensitivity

A 20 % (*v/v*) ammonia solution was employed to simulate an alkaline environment (meat spoilage), and glacial acetic acid (99.5 %, *v/v*) was used to replicate an acidic environment (fruit/vegetable spoilage) (Zhang et al., 2022). The dual-function film was subjected to alternating exposure to both vapors to evaluate its pH-responsive reversibility. The film was placed in a 100 mL conical flask containing 20 mL of ammonia solution (20 %, *v/v*) for 20 min. Chromaticity changes were recorded using a digital camera at fixed intervals, and color parameters (L*, a*, b*) were measured with a chroma meter. *ΔE* values were computed as shown in Eq. (5), using the initial film color as the baseline. After 10 min, the film was moved to a headspace vial with glacial acetic acid (99.5 %, *v/v*) for 15 min, and color changes were again documented. This procedure was repeated, with the film being re-exposed to ammonia vapor, to assess the reversible response of the film under alternating acidic and basic conditions.

#### 2.4.12 UV–vis wavelength transmittance and opacity

The film was trimmed into rectangular strips that corresponded to the test tube sidewall dimensions and firmly adhered to its surface. Using a UV–vis spectrophotometer (UV-2600, Shimadzu, Kyoto, Japan), the transmittance of the film was measured across the 350–800 nm range, and absorbance at 600 nm was recorded. To calculate opacity, the following formula was used:

(6) $$\begin{array}{c} \mathrm{Opacity}=\mathrm{Abs}600/d\# \end{array}$$

where Abs600 is the absorbance value of the film at 600 nm and d is the thickness of the film in mm.

#### 2.4.13 Evaluation of antioxidant activity

The DPPH radical scavenging activity of the films was determined using a modified procedure (Longwei et al., 2022). Film samples weighing 4, 8, 12, 16, and 20 mg were mixed with 3 mL of DPPH solution, vortexed, and the absorbance at 517 nm was measured using a UV–Vis spectrophotometer (UV-2600, Shimadzu, Kyoto, Japan).

Similarly, the ABTS radical scavenging activity was assessed using a modified method (Longwei et al., 2022). Film samples of 4, 8, 12, 16, and 20 mg were combined with 3 mL of ABTS solution, vortexed, and the absorbance at 734 nm was recorded using a UV–Vis spectrophotometer (UV-2600, Shimadzu, Kyoto, Japan).

The radical scavenging rate was calculated using the formula:

(7) $$\begin{array}{c} \mathrm{Free radicals cavengingrate}\left(\%\right)=\frac{A_{0}-A_{1}}{A_{0}}\times 100\# \end{array}$$

where A_0_ is the absorbance value of the blank at 517 nm or 734 nm and A_1_ is the absorbance value of the sample at 517 nm or 734 nm.

#### 2.4.14 Antimicrobial activity

A 1 mL aliquot of *S. aureus* and *E. coli* bacterial suspensions (∼10^6^ CFU/mL) was spread homogeneously on solid agar plates. The film specimens were fixed onto the inner lid of the Petri dishes, sealed with parafilm, and incubated at 37 °C for 18 h.

#### 2.4.15 Release of CL

The release of essential oils from the IC-FA-AMA/PS I films was studied using three different food simulants, following a modified procedure (Zhang et al., 2022). Distilled water was used to simulate aqueous food, 50 % (*v/v*) ethanol solution to simulate oil-in-water emulsions and alcoholic food, and 95 % (*v/v*) ethanol to simulate fatty food. The films were agitated at 60.5 ×g using an orbital shaker at 25 °C. Every 12 h, 0.1 mL of the solution was extracted, diluted tenfold with ethanol, and the absorbance at 277 nm was measured with a UV–Vis spectrophotometer. Essential oil concentrations were determined from a pre-established calibration curve. This process was repeated until a release equilibrium was established, and the CL release profile was generated.

Furthermore, referring to the method of Song, Hou, et al. (2024), the Ritger- Peppas model was used to evaluate the release of CL from the active intelligent film.

(8) $$\begin{array}{c} \frac{M_{t}}{M_{\infty }}=kt^{n}\# \end{array}$$

where M_t_ is the amount of CL in food simulants at t time; M_∞_ is the equilibrium amount of CL in food simulants; k is the release rate constant; t is the release time; n represents the release mechanism index of CL in the film.

### 2.5 Molecular docking simulation

Molecular docking studies of FA, cyanidin-3-O-galactoside (the primary component of AMA) (Chen et al., 2024), CL, HP-β-CD, and PS with HP-β-CD were conducted using AutoDock Vina. Due to the structural variation of starch molecules, a model of linear α-1,4-D-glucan with a degree of polymerization of 10 (DP = 10, Sigma-Aldrich, analytical standard) was chosen for amylose. Topology construction began with the chemical structure design software, King Draw v3.2.1 (Chem Bean Soft), followed by 3D conformation optimization through the Yin Fu Tech Molecular Cloud Platform (https://cloud.yinfotek.com) (Yinfotek Co., L, 2025). The 3D structures of other compounds were sourced from the PubChem database, with the CIDs as follows: FA: 445858; cyanidin-3-O-galactoside: 441699; CL: 0364; HP-β-CD: 14049689. Format conversion was carried out using Open Babel GUI. Molecular docking results were visualized with PyMol.

### 2.6 Shrimp-freshness monitoring

The method described by Chen et al. (2024) was adapted for this study. Shrimps, each weighing approximately 25 g, were thoroughly rinsed with deionized water and dried to remove surface moisture. The shrimps were then divided into five groups for packaging treatment: (1) control group (unpackaged); (2) shrimps cover with IC-FA-AMA/PS I film. The samples were stored at 4 °C for 60 h, with measurements taken at the following time intervals: 0, 12, 24, 36, 48, 60, and 72 h. The pH of the shrimp samples was determined according to the Chinese National Standard GB 5009.237–2016 (China, N. H. C. o. t. P. s. R. o. C. S. A. o. t. P. s. R. o, 2016a). The total viable count (TVC) was measured following the procedures outlined in GB 4789.2–2022 (China & o. t. P. s. R. o. C. S. A. o. t. P. s. R. o., 2022). The volatile basic nitrogen (TVB-N) content was analyzed according to GB 5009.228–2016 (China, N. H. C. o. t. P. s. R. o. C. S. A. o. t. P. s. R. o, 2016b).

### 2.7 Statistical analyses

Each experiment was performed in triplicate. The results are presented as the mean ± standard deviation (SD). Statistical evaluations were carried out using analysis of variance (ANOVA) in IBM SPSS Statistics software (IBM Corp., Armonk, NY, USA). Duncan's multiple range test was employed to identify significant differences between the means at *P* < 0.05. Graphical data presentation was achieved using Origin 2018 software (Origin Lab Co., Northampton, MA, USA).

## 3 Results and discussion

### 3.1 Copigmentation facilitated pH sensitivity

**Fig. 1:**
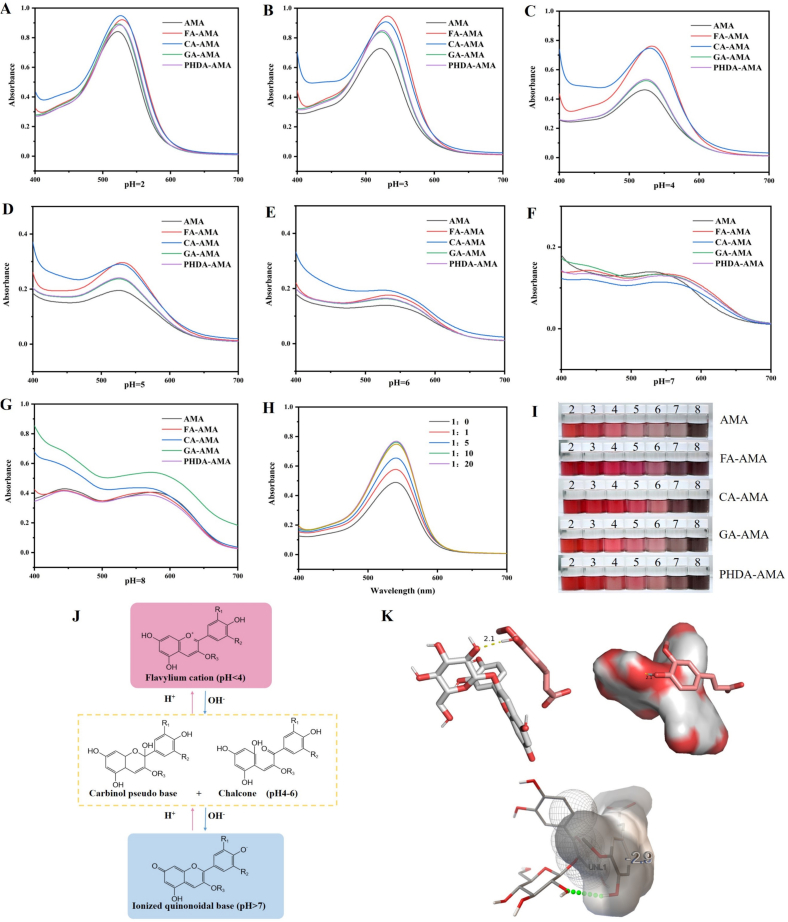
pH 2–8 UV–vis spectra (A-G), UV–vis spectra of AMA cochromes with different molar ratios of FA (H), color changes of anthocyanin solutions and their solutions with four phenolic acids at different pH values (I), structural transformations of anthocyanins (J), molecular docking simulations of the main component of AMA, cornflavin-3-O-glucoside, and FA (K).(AMA: *Aronia melanocarpa* anthocyanins solutions; FA-AMA: Copigment solution of ferulic acid and anthocyanins; CA-AMA: Copigment solution of caffeic acid and anthocyanins; GA-AMA: Copigment solution of gallic acid and anthocyanins; PHDA-AMA: Copigment solution of Hydroxybenzoic acid and anthocyanins.)

**Scheme 1:**
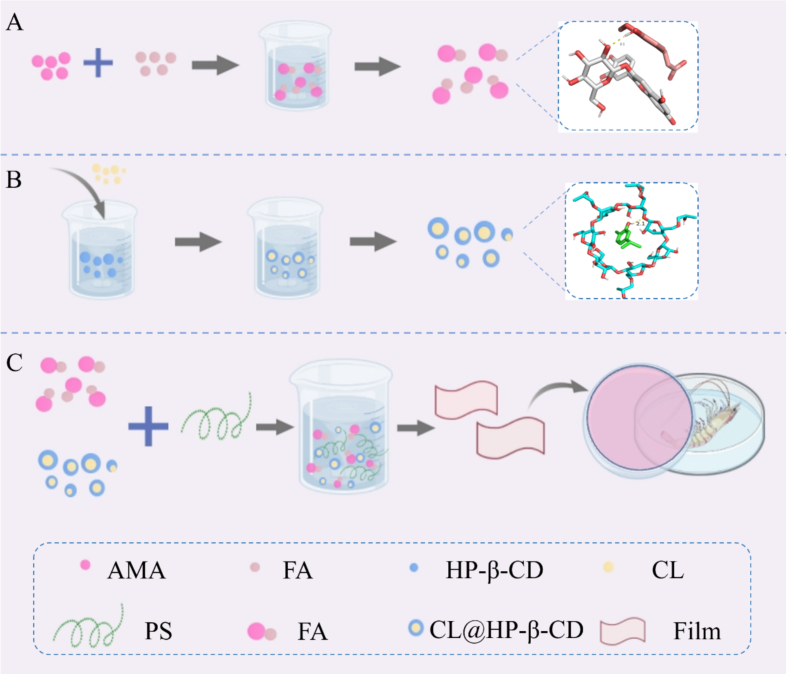
FA copigmented AMA (A); HP-β-CD encapsulated CL (B); Preparation of film and preservation of shrimp (C).

The UV–Vis spectra (Fig. 1A-G) demonstrate that the four phenolic acids (FA, CA, GA, PHDA) enhance both the maximum absorption wavelength and intensity of AMA, and this behavior is attributed to copigmentation, which leads to a bathochromic shift and hyperchromic effect (Amico et al., 2004). It has been reported that the benzene ring of phenolic acid and the conjugated system of AMA interact through π–π stacking, preventing the formation of a colorless methanol pseudo-base and preserving the red hue of anthocyanins (Guo et al., 2021). The pH range of 4–6 was identified as crucial for the response sensitivity of the film, with FA-AMA exhibiting the most significant redshift (Fig. 1C-E), suggesting that the copigmented AMA has undergone structural changes, likely driven by hydrogen bonding between the hydroxyl groups of FA and the hydroxyl, carbonyl and other groups in AMA (Crupi, 2023), thereby enhancing the color stability of AMA. The pH-dependency of phenolic acid copigmentation effects was observed (Fig. 1H), with FA-AMA showing the greatest impact, aligning with the UV–Vis data. Molecular docking simulations confirmed a hydrogen bonding interaction (2.1 Å) between cyanidin-3-O-galactoside and FA (Fig. 1K), with a binding energy of −2.9 kcal/mol, strengthening the FA-AMA binding and improving both AMA color intensity and copigmentation efficiency. (See Scheme 1.)

An increase in FA concentration led to a greater formation of copigmentation complexes with AMA, which in turn stabilized their structure and boosted the color intensity, resulting in a higher UV–Vis absorption. However, when the AMA to FA molar ratio surpassed 1:10, further additions of FA had minimal impact on the UV absorption intensity, and this is likely because FA reached saturation in occupying the available binding sites on AMA molecules (Singh et al., 2025). These observations are consistent with earlier research on the copigmentation-driven stabilization of *Vitis vinifera* anthocyanins at varying molar ratios (Lv et al., 2022).

### 3.2 Preparation and characterization of IC

#### 3.2.1 SEM and energy dispersive spectroscopy (EDS) of IC

**Fig. 2:**
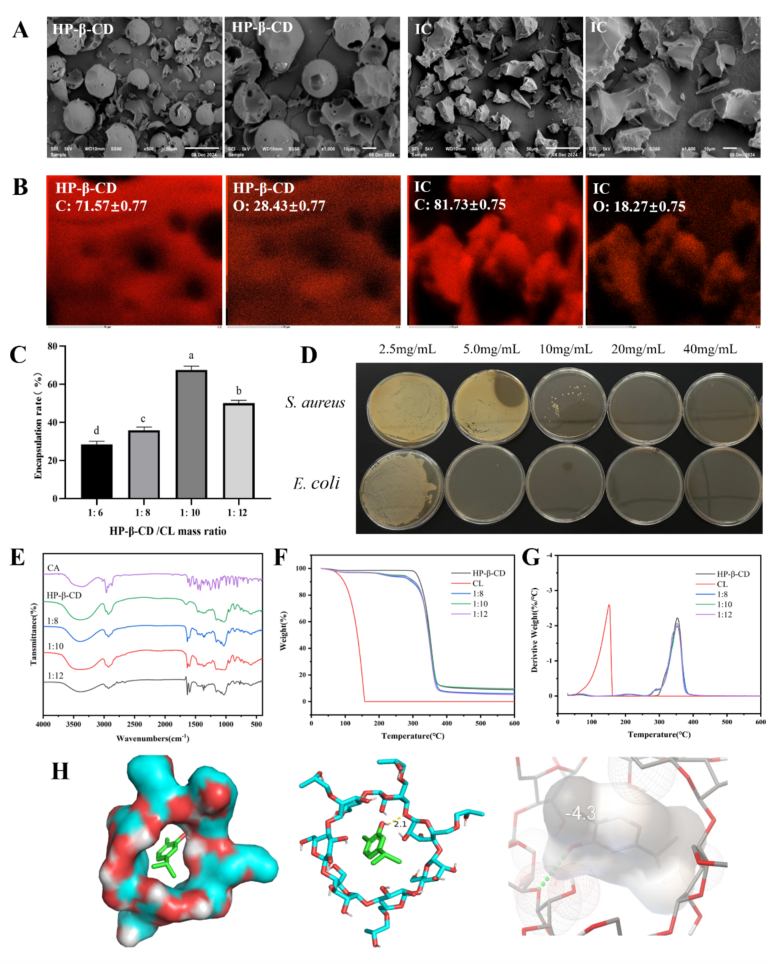
SEM (A) and EDS (B) images of HP-β-CD and IC, IC embedding ratio for different wall-core mass ratios (C), Bactericidal concentration of IC (D), FT-IR spectra (E), TGA curves (F) and DTG curves (G), CL and HP-β-CD molecular docking simulations (H). (HP-β-CD: hydroxypropyl-β-cyclodextrin; IC: Hydroxypropyl-β-cyclodextrin microcapsules filled with carvacrol.)

SEM analysis (Fig. 2A) indicated that HP-β-CD displayed a spherical structure with prominent cavity formations, while the IC presented a flatter and more irregular, block-like appearance. This alteration in morphology implies the successful incorporation of CL into the cavities of HP-β-CD, forming a novel composite phase and modifying the surface structure of IC. These results are consistent with earlier studies on HP-β-CD-encapsulated fennel essential oil (Song, Lu, et al., 2024). EDS data (Fig. 2B) further revealed a notable shift in the carbon/oxygen (C/O) atomic ratio after CL encapsulation within HP-β-CD, supporting the conclusion that CL is effectively trapped within the HP-β-CD matrix. A similar C/O ratio change was observed in (Ates & Yildiz, 2025) study on the encapsulation of CL by β-cyclodextrin-based metal-organic frameworks, where carbon content increased and oxygen content decreased.

#### 3.2.2 Embedding rate of IC

As shown in Fig. 2C, the encapsulation efficiency of CL increased with the mass ratio of HP-β-CD to CL, peaking at 68 % when the ratio reached 1:10 (*w/w*). After surpassing this ratio, a decrease in encapsulation efficiency was observed, likely due to the saturation of available binding sites in the HP-β-CD cavities, preventing further efficient encapsulation of excess CL and resulting in lower entrapment efficiency (Rao et al., 2022). These findings align with the research results of Ertan et al. (2023) on the encapsulation of carvacrol by γ-cyclodextrin.

#### 3.2.3 FTIR of IC and molecular docking simulation

As depicted in Fig. 2E, the peak at 3448 cm^−1^ in the spectrum of CL is attributed to the stretching vibration of the −OH group (Lu et al., 2025). Sharp and intense peaks observed at 1625 and 1675 cm^−1^ correspond to the C − H deformation of the benzene ring and olefinic groups, while the peaks at 747 and 688 cm^−1^ are related to the out-of-plane bending vibrations of aromatic C − H bonds. HP-β-CD displayed a broad absorption between 3540 and 3220 cm^−1^, likely due to the −OH stretching vibration. Additional characteristic peaks for HP-β-CD were observed at 2924 cm^−1^ for C − H stretching and at 1650 cm^−1^ for H − O − H bending (Zhe et al., 2022). After encapsulation, the characteristic peaks of CL became undetectable, and the broad absorption between 3540 and 3220 cm^−1^ expanded, with enhanced intensity at 1650 cm^−1^. These changes in the spectrum indicates that CL was encapsulated within HP-β-CD, likely due to hydrogen bonding interactions between the two. These results are in agreement with the findings reported by Zhe et al. (2022). The molecular docking simulations of CL and HP-β-CD (Fig. 2H) further confirmed these interactions. Hydrogen bonding and hydrophobic interactions were observed, with the hydrogen bond distance between CL and HP-β-CD measured at 2.1 Å and the binding energy calculated at −4.3 kcal/mol. These interactions enabled successful encapsulation of CL in the cavity of HP-β-CD, contributing to enhanced stability.

#### 3.2.4 Thermal stability of IC

As seen in Fig. 2F, CL exhibited a single weight loss step between 30 and 157 °C, corresponding to thermal volatilization of CL. Both HP-β-CD and its IC showed initial mass losses at 40–100 °C, attributed to evaporation of residual moisture from the cyclodextrin cavity. The second decomposition stage occurred at 290–395 °C. The peak decomposition rate for HP-β-CD occurred at 350 °C, resulting in an 83 % mass loss (Fig. 2G). The IC exhibited a weight loss in the range of 160 to 250 °C, indicative of free CL volatilization (Zhe et al., 2022). The derivative thermogravimetric analysis (DTG) curve highlighted an increase in decomposition rates between 290 and 300 °C, due to the disruption of CL-HP-β-CD hydrogen bonds at elevated temperatures, leading to the volatilization of CL (Celebioglu & Uyar, 2021). This observation aligns with previous findings by Celebioglu and Uyar (2021), where the volatilization temperature of eugenol shifted from 190 to 300 °C upon complexation with γ-CD. Taken together, these results suggest that HP-β-CD effectively encapsulates CL within its hydrophobic cavity, enhancing its thermal stability through a combination of hydrogen bonding and hydrophobic interactions.

#### 3.2.5 Antimicrobial properties of IC

The IC demonstrated bacteriostatic effects against both *E. coli* and *S. aureus*, with a clear positive correlation between its activity and the CL concentration. The antibacterial activity of carvacrol against *E. coli* and *S. aureus* is attributed to its distinct mechanisms of action. On one hand, against *E. coli*, carvacrol initially disrupts the outer membrane and subsequently compromises the cytoplasmic membrane, ultimately leading to cell lysis (Jiménez et al., 2018). On the other hand, for *S. aureus*, it directly interacts with the peptidoglycan layer, interfering with its biosynthesis and impairing cell wall integrity, thereby conferring potent bactericidal effects (dos Santos Rodrigues et al., 2017). The MICs for *E. coli* and *S. aureus* were found to be 5 and 10 mg/mL, respectively, while the MBCs for these bacteria were 10 and 20 mg/mL, respectively. These results suggest that the IC exhibits stronger inhibitory effects on *E. coli* compared to *S. aureus*, and this observation aligns with the findings of Majeed et al. (2023).

### 3.3 Characterization of films

#### 3.3.1 Morphological of films

**Fig. 3:**
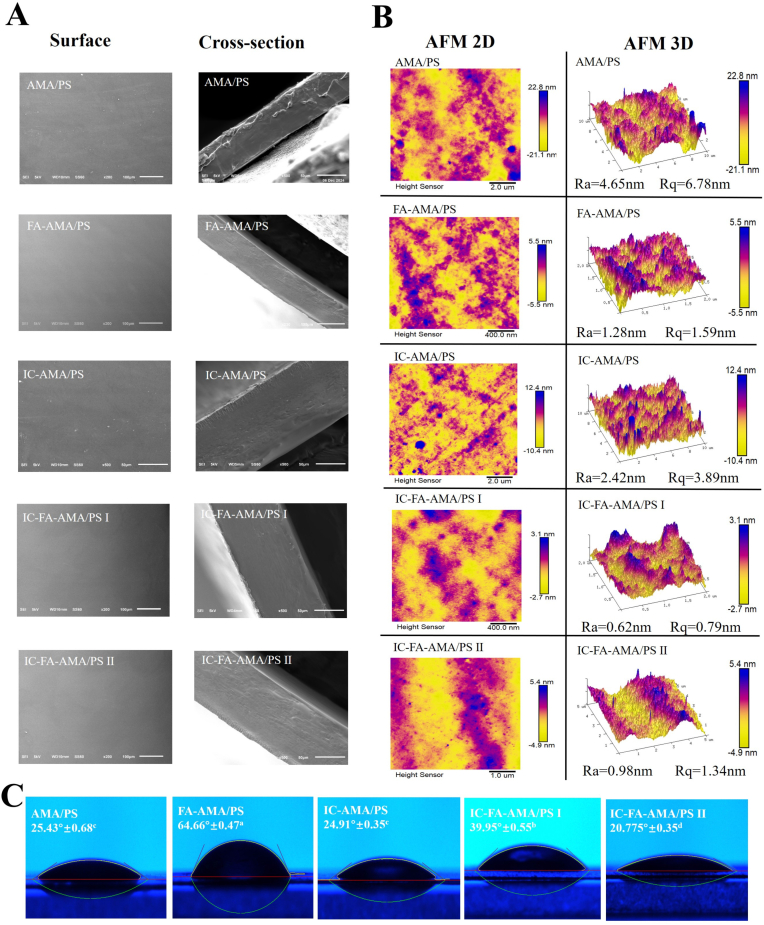
SEM (A), AFM (B) and WCA (C) images of films. Different letters in the same column indicate significantly different (*p* < 0.05). (AMA/PS: *Aronia melanocarpa* anthocyanins/potato starch film; FA-AMA/PS: *Aronia melanocarpa* anthocyanins /potato starch film with ferulic acid; IC-AMA/PS: *Aronia melanocarpa* anthocyanins/potato starch film with 20 mg/mL IC; IC-FA-AMA/PS I: Ferulic acid-*Aronia melanocarpa* anthocyanins/potato starch film with 20 mg/mL IC; IC-FA-AMA/PS II: Ferulic acid-*Aronia melanocarpa* anthocyanins/potato starch film with 40 mg/mL IC.)

SEM analysis (Fig. 3A) demonstrated that films without FA (AMA/PS, IC-AMA/PS) exhibited granular morphology on both their surface and cross-section, while AFM measurements (Fig. 3B) revealed significantly higher Ra and Rq values. In contrast, FA-modified films (FA-AMA/PS, IC-FA-AMA/PS I, IC-FA-AMA/PS II) showed smoother surfaces and more homogeneous cross-sectional structures, which was also reflected in the lower Ra and Rq values in AFM. These findings likely due to hydrogen bonding between FA and AMA, and such interactions lead to improved molecular packing and structural organization, resulting in smoother film surfaces (Wang et al., 2023).

When IC was incorporated into FA-AMA/PS films (IC-FA-AMA/PS I, IC-FA-AMA/PS II), a further reduction in Ra and Rq values was noted, indicating excellent compatibility between IC and the film matrix, which may also be attributed to non-covalent interactions, such as hydrogen bonding between IC and PS. In contrast, higher concentrations of IC led to an increase in Ra and Rq values, which may lie in the tendency of excess IC to aggregate, and these aggregates are difficult to disperse uniformly during film formation, resulting in increased surface roughness (Bansal et al., 2025). Chen et al. (2024) prepared a film by stabilizing AMA with tea polyphenols, and their AFM results showed Ra = 1.29 and Rq = 1.28. In contrast, the AFM results of the IC-FA-AMA/PS I film prepared in this study revealed Ra = 0.62 and Rq = 0.79, indicating a smoother film surface.

#### 3.3.2 Physical properties

The thickness of the films showed a tendency to increase with the solid matrix content, but the change was not statistically significant (Table 1), which aligns with the SEM observations.

**Table 1:** Thickness, WVP, TS and EB of films.

Films	Thinkness (mm)	WVP 10^−10^ (g/m.s.Pa)	TS (MPa)	EB (%)
AMA/PS	0.087 ± 0.009^a^	3.87 ± 0.33^a^	1.01 ± 0.93^a^	32.85 ± 1.93^a^
FA-AMA/PS	0.095 ± 0.018^a^	3.71 ± 0.61^a^	1.19 ± 1.61^a^	48.36 ± 2.13^b^
IC-AMA/PS	0.104 ± 0.022^a^	3.06 ± 0.42^b^	1.97 ± 0.88^b^	89.41 ± 1.67^c^
IC-FA-AMA/PS I	0.115 ± 0.053^a^	2.95 ± 0.54^b^	2.06 ± 1.04^b^	103.82 ± 2.48^d^
IC-FA-AMA/PS II	0.129 ± 0.034^a^	2.85 ± 0.12^b^	2.10 ± 0.52^b^	111.14 ± 3.15^e^

Note: Different letters in the same column indicate significantly different (*p* < 0.05).

As presented in Table 1, the addition of FA did not lead to a significant change in the WVP of the films. However, incorporating IC into both AMA/PS and FA-AMA/PS films notably reduced the WVP (*p* < 0.05), and this decrease is likely due to hydrogen bonding interactions between the HP-β-CD groups of IC and the hydroxyl groups of PS. Such interactions enhance the internal chain forces of the film matrix, leading to greater film compactness (Li et al., 2023). When the IC concentration exceeded optimal levels, no significant reduction in WVP was observed, likely because the hydrogen bonding interactions became saturated with the excessive addition of IC (Li, Wu, et al., 2025). This observation is consistent with the findings of Li, Wu, et al. (2025) on the incorporation of microcapsules into PS/sodium alginate composite films.

The addition of FA to the AMA/PS film led to a significant rise in the EB (*p* < 0.05) (Table 1), likely due to the weakening of the rigid structural framework of the film (Ma & Wang, 2016). After incorporating IC into the films, a notable increase in both TS and EB with the increase in IC concentration (*p* < 0.05). The enhanced film performance may be due to the intercalation of HP-β-CD between starch chains, leading to greater film compactness, improved flexibility, and better elasticity, which helps distribute applied stresses and increase EB (Li et al., 2023). This observation aligns with the results from Shen et al. (2022) on the use of HP-β-CD inclusion complexes in microfilms. Based on the above conclusions, further improvements in the TS of the films are necessary.

As shown in Fig. 3C, the addition of FA to AMA/PS films led to a notable rise in WCA (*p* < 0.05). This improvement can be attributed to hydrogen bonds formed between FA and AMA, which decrease the availability of surface hydroxyl groups, thus enhancing the film hydrophobicity (Hao et al., 2023). The inclusion of IC in the AMA/PS films also resulted in an increased WCA, likely due to interactions between the hydroxyl groups on the outer cavity of HP-β-CD and those in PS that further reduce the exposure of hydroxyl groups (Zheng et al., 2023). When both FA and IC were added together (IC-FA-AMA/PS I), a synergistic effect was observed. However, when the IC concentration was too high, the WCA decreased, possibly due to the increased presence of free hydroxyl groups from unbound IC molecules.

#### 3.3.3 The crystalline structure analysis of films

**Fig. 4:**
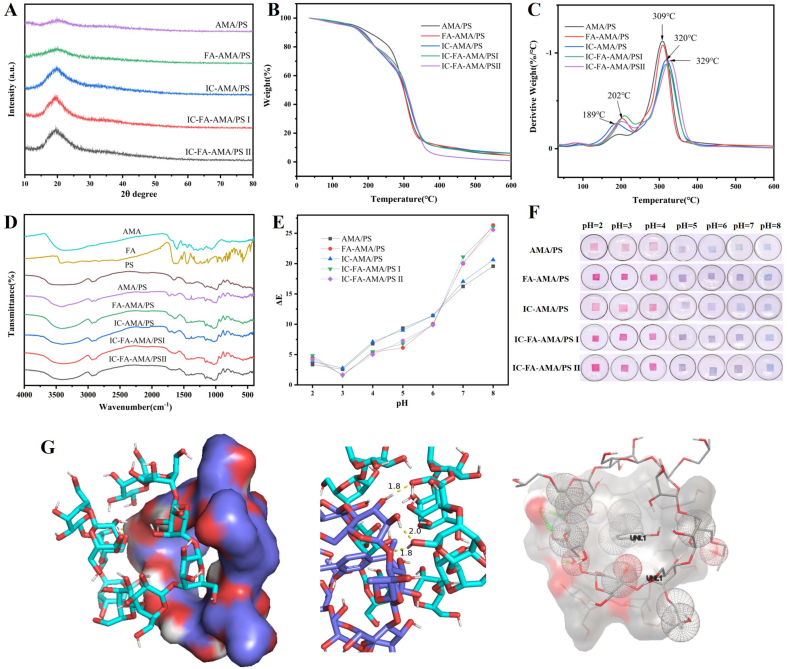
XRD pattern (A), TGA curve (B) and DTG curve (C), FTIR spectra (D) of the films, *ΔE* values (E) and color response (F) of films in solution conditions of pH 2–8, molecular docking simulation between PS and HP-β-CD (G). (AMA/PS: *Aronia melanocarpa* anthocyanins/potato starch film; FA-AMA/PS: *Aronia melanocarpa* anthocyanins /potato starch film with ferulic acid; IC-AMA/PS: *Aronia melanocarpa* anthocyanins/potato starch film with 20 mg/mL IC; IC-FA-AMA/PS I: Ferulic acid-*Aronia melanocarpa* anthocyanins/potato starch film with 20 mg/mL IC; IC-FA-AMA/PS II: Ferulic acid-*Aronia melanocarpa* anthocyanins/potato starch film with 40 mg/mL IC.)

The XRD patterns presented in Fig. 4A highlight the compatibility of materials and the intermolecular interactions within the films. All groups exhibited a diffraction peak at 2θ = 20°. Upon incorporating FA into the AMA/PS film, the diffraction peak intensity increases because hydrogen bonding between FA and AMA modified the molecular interactions and arrangement. The addition of IC further enhanced the peak intensity, which can be attributed to two factors: firstly, IC influenced the regularity of molecular chain alignment and the crystallization process (Klonos et al., 2024); secondly, IC likely formed additional interactions with PS. Notably, no new diffraction peaks were observed following the IC incorporation, confirming that the film components remained compatible and that IC did not alter the crystalline structure (Chen et al., 2024). When both FA and IC were incorporated, the peak intensity increases more remarkably due to the combined effects of hydrogen bonding between FA and AMA and the new interactions formed between IC and PS (Stevens et al., 2013).

#### 3.3.4 FTIR of films and molecular docking simulation

The FTIR analysis of FA (Fig. 4D) revealed significant peaks at 3433 and 1620 cm^−1^, corresponding to −OH stretching and C=O stretching, respectively, with aromatic ring vibrations observed in the range of 1500–700 cm^−1^. AMA displayed peaks at 3428, 1635, 1314, and 1071 cm^−1^, corresponding to −OH stretching, C=C stretching, C − O bending, and glycosidic linkages (Qin et al., 2021). PS displayed absorption peaks at 3444 cm^−1^ for −OH stretching vibration, 2934 cm^−1^ for C − H stretching vibration, 1650 cm^−1^ for −OH bending vibration, and 1004 cm^−1^ for C − O stretching vibration (Wang et al., 2019). When FA was incorporated into the AMA/PS film, a noticeable red-shift of the −OH peaks occurred, indicating the formation of hydrogen bonds between FA and AMA. The addition of IC resulted in shifts of all −OH peaks and increased their intensity, while −CH and C − O bond vibrations shifted to 2931 and 1031 cm^−1^, respectively, with sharper peaks. This suggests altered intermolecular forces, likely due to the formation of hydrogen bonds between HP-β-CD and PS. This observation aligns with the infrared analysis by Li et al. (2023) on β-cyclodextrin and PS. In this study, the interaction mechanism between PS and HP-β-CD was innovatively revealed through molecular docking simulations. Fig. 4G shows that hydrogen bonds between PS and HP-β-CD with bond lengths of 1.8, 1.8, and 2.0 Å and a binding energy of −4.4 kcal/mol. Hydrophilic interactions were also noted between PS and HP-β-CD, which contributed to a more stable structure and enhanced the film properties (Hu et al., 2024).

#### 3.3.5 Thermal stability of films

The films underwent thermal degradation in three distinct phases (Fig. 4B-C). In the first phase (60–100 °C), water evaporation caused a 3–4 % mass loss. The second phase, occurring at 150–240 °C, involved the degradation of AMA. FA-free films showed the highest mass loss at 189 °C, while FA-modified films exhibited a shift in the degradation peak, suggesting that FA-AMA hydrogen bonds enhanced thermal stability. In the third phase (250–350 °C), PS decomposition occurred, leading to chain scission and structural breakdown (Li et al., 2023). For films without IC, the peak mass loss occurred at 309 °C, whereas incorporating IC shifted this peak to 320 °C, likely due to stabilizing interactions between PS and HP-β-CD. Cui et al. (2023) also demonstrated in their study on mesoporous nano-silica PS films that hydrogen bonding between PS and HP-β-CD contributed to improved thermal stability. In this study, molecular docking simulations confirmed the existence of hydrogen bonding and hydrophilic interactions between PS and HP-β-CD (Fig. 4G).

#### 3.3.6 Storage color stability of films

Films with FA (FA-AMA/PS, IC-FA-AMA/PS I, IC-FA-AMA/PS II) show a noticeable increase in color intensity compared to their FA-free equivalents (AMA/PS, IC-AMA/PS) (Fig. S1). Under the same temperature conditions, the FA-modified films showed a lower *ΔE* value after 30 d compared to the non-FA films, indicating that FA enhances color stability. This supports the role of FA in both enhancing and stabilizing the color of AMA, likely due to hydrogen-bonding interactions between FA and AMA, which prevents the hydroxyl groups in AMA from being oxidized to quinones and decomposing into colorless products (Vázquez-González et al., 2025). Interestingly, the incorporation of IC had a minimal impact on the coloration of the films.

#### 3.3.7 Color response of films to pH changes

The pH-responsive chromatic changes of the films (FA-AMA/PS, IC-FA-AMA/PS I, IC-FA-AMA/PS II) are shown in Fig. 4F. As the pH increased from 2 to 8, a shift from red to blue-green colors occurred (Chayavanich et al., 2023). The films containing FA exhibited more intense color and clearer spectral differentiation compared to those without FA, particularly between pH 4 and 5. This was accompanied by a wider range of *ΔE* values (Fig. 4E). The increased chromatic sensitivity confirms that FA enhances the pH-responsiveness of the films. FA hydroxyl groups interact with those in AMA, preventing the formation of colorless methanol pseudo bases in anthocyanins (Guo et al., 2021), thus improving the stability of AMA.

#### 3.3.8 Reversible color response of films

**Fig. 5:**
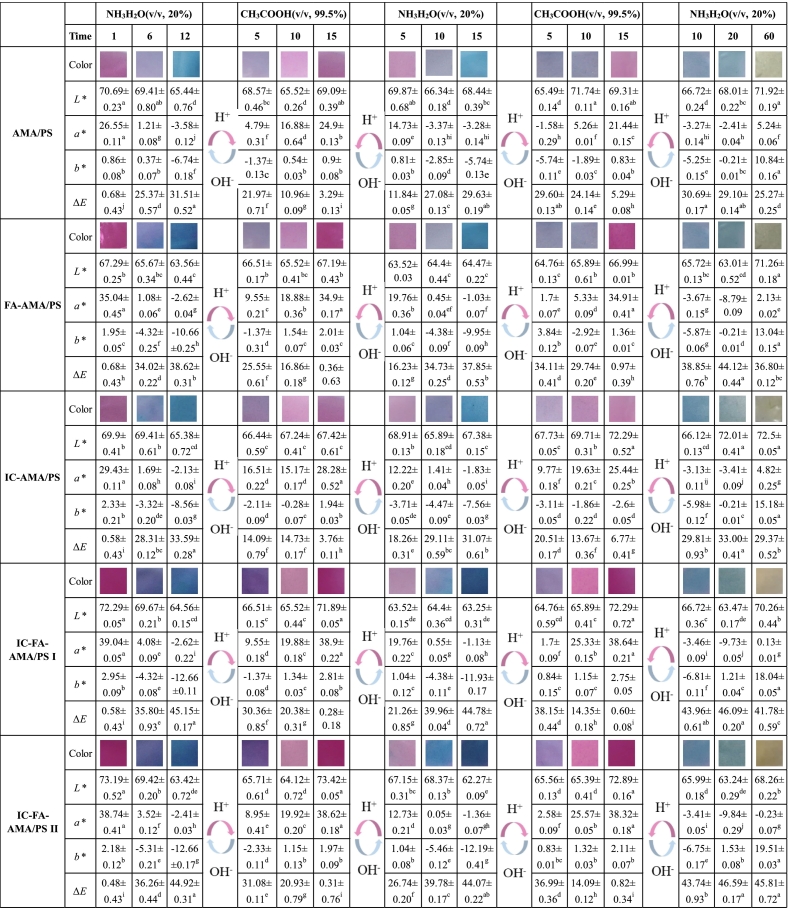
Changes in color and L*, a*, b*, Δ*E* values during acid-base response of films. (AMA/PS: *Aronia melanocarpa* anthocyanins/potato starch film; FA-AMA/PS: *Aronia melanocarpa* anthocyanins /potato starch film with ferulic acid; IC-AMA/PS: *Aronia melanocarpa* anthocyanins/potato starch film with 20 mg/mL IC; IC-FA-AMA/PS I: Ferulic acid-*Aronia melanocarpa* anthocyanins/potato starch film with 20 mg/mL IC; IC-FA-AMA/PS II: Ferulic acid-*Aronia melanocarpa* anthocyanins/potato starch film with 40 mg/mL IC.)

The acid-base chromogenic reversibility of the films is illustrated in Fig. 5. With increasing exposure time to ammonia, the a* value of the films gradually decreased, while the b* value and *ΔE* value increased progressively, accompanied by a distinct color change from pink to blue. Subsequent exposure to glacial acetic acid, the a* value increased gradually, whereas the b* and *ΔE* values decreased, leading the films to revert from blue to their initial red color. After undergoing two ammonia/acetic acid cycles, the films without FA exhibited significant changes in *ΔE*, L*, a*, and b* values compared to the initial state. In contrast, the *ΔE*, L*, a*, and b* values of the FA-incorporated films showed no significant variations, and their color remained nearly identical to the original state. This indicates that the films maintained a robust color response even after repeated exposure to acidic and alkaline environments, with no impairment of their photosensitivity and sensitivity. In contrast, the FA-free films could hardly revert to the original red color during the second cycle, and the yellow-green color displayed after prolonged exposure to ammonia was not distinct. These phenomena can be attributed to the formation of hydrogen bonding interactions between FA and cyanidin-3-O-galactoside in AMA. This interaction stabilizes the molecular structure of anthocyanins, inhibits the transformations of anthocyanins to the colorless chalcone pseudo bases and chalcone form (Li, Yang, et al., 2025). In comparison to Li et al. (2023), who studied the single-reversible color response of blueberry anthocyanins with FA copigmentation, the current study demonstrates superior acid-base reversibility and sensitivity in the active intelligent films after two cycling experiments.

#### 3.3.9 UV–vis wavelength transmittance and opacity

**Fig. 6:**
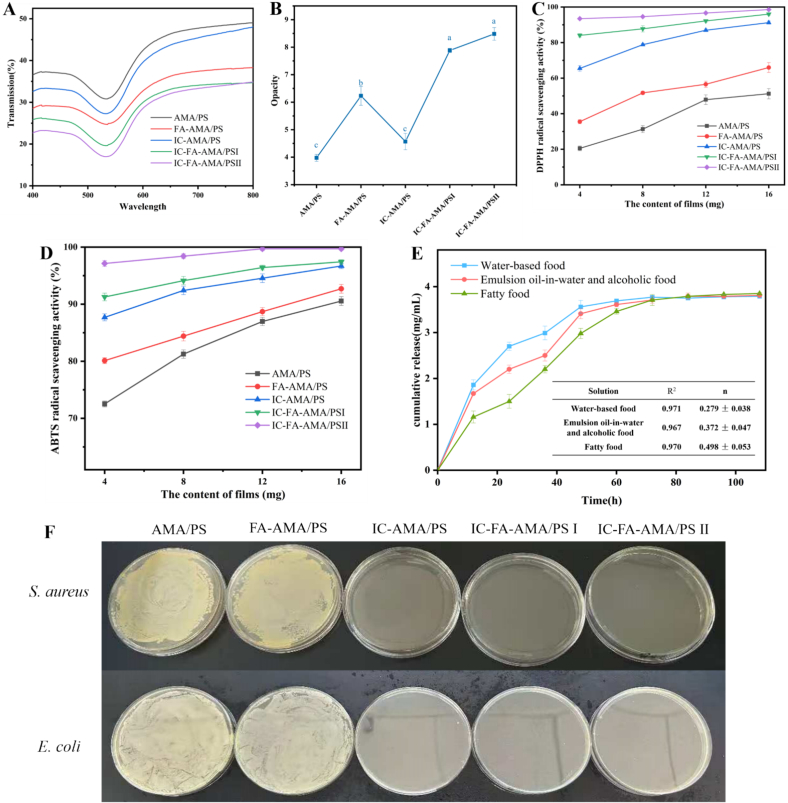
UV transmittance of films (A) and opacity (B), DPPH scavenging activity (C) and ABTS scavenging activity (D), Slow-release curves of IC-FA-AMA/PS I in different food simulants, correlation coefficients R^2^ and release exponents (n) for the Ritger-Peppas model (E), the bacteriostatic effect of films against *E. coli* and *S. aureus* (F). Different letters in the same column indicate significantly different (*p* < 0.05). (AMA/PS: *Aronia melanocarpa* anthocyanins/potato starch film; FA-AMA/PS: *Aronia melanocarpa* anthocyanins /potato starch film with ferulic acid; IC-AMA/PS: *Aronia melanocarpa* anthocyanins/potato starch film with 20 mg/mL IC; IC-FA-AMA/PS I: Ferulic acid-*Aronia melanocarpa* anthocyanins/potato starch film with 20 mg/mL IC; IC-FA-AMA/PS II: Ferulic acid-*Aronia melanocarpa* anthocyanins/potato starch film with 40 mg/mL IC.)

After the incorporation of FA into the AMA/PS and IC-AMA/PS films led to a significant decrease in UV transmittance (Fig. 6A) and a notable increase in opacity (Fig. 6B). These results indicate that the addition of FA effectively enhances the color stability of the films. In contrast, the incorporation of IC into the AMA/PS and FA-AMA/PS films resulted in only minor reductions in UV transmittance and opacity, with these changes not reaching statistical significance. This can be attributed to two factors: First, the addition of FA promotes the formation of flavylium cations in AMA, which intensifies the red hue. Notably, flavylium cations contain multiple conjugated benzene rings, which contribute to the enhanced UV barrier properties of the films, while the deepened red hue further increases opacity (Cao et al., 2023). Second, the hydroxycinnamic acid moiety in the FA structure provides strong UV absorption capacity (Horbury et al., 2016). Similar findings have been documented in the work of Zhan et al. (2025).

#### 3.3.10 Antimicrobial properties

As shown in Fig. 6F, significant bacterial growth was observed for both *S. aureus* and *E. coli* on petri dishes covered with films that did not contain IC (AMA/PS, FA-AMA/PS). In contrast, the films containing IC (IC-AMA/PS, IC-FA-AMA/PS I, IC-FA-AMA/PS II) exhibited clearer petri dishes with no visible bacterial colonies, indicating that the CL in IC is released through volatilization, providing the films with effective antibacterial properties. This finding is consistent with previous research on CL-loaded Pickering emulsion gelatin films, which similarly demonstrated inhibitory effects against *S. aureus* and *E. coli* (Liu, Wang, et al., 2024). These results highlight the potential of the developed active intelligent films to extend food shelf life in controlled environments.

#### 3.3.11 Antioxidant properties of films

Incorporating FA into the FA-AMA/PS films significantly boosted their ABTS (Fig. 6D) and DPPH radical (Fig. 6C) scavenging capacity, due to the capability of FA to donate hydrogen atoms from phenolic groups, thereby enhances radical scavenging activity (Zduńska et al., 2018). Likewise, the addition of IC to the films (IC-AMA/PS, IC-FA-AMA/PS I, IC-FA-AMA/PS II) greatly increased the scavenging of ABTS and DPPH radicals, owing to the antioxidant properties of the encapsulated CL. The combined inclusion of FA and IC resulted in a synergistic enhancement of the films' antioxidant properties, with an increase in activity directly corresponding to higher IC concentrations. These results underscore the strong antioxidant capabilities of the active intelligent films developed in this study.

#### 3.3.12 Release of CL

**Fig. 7:**
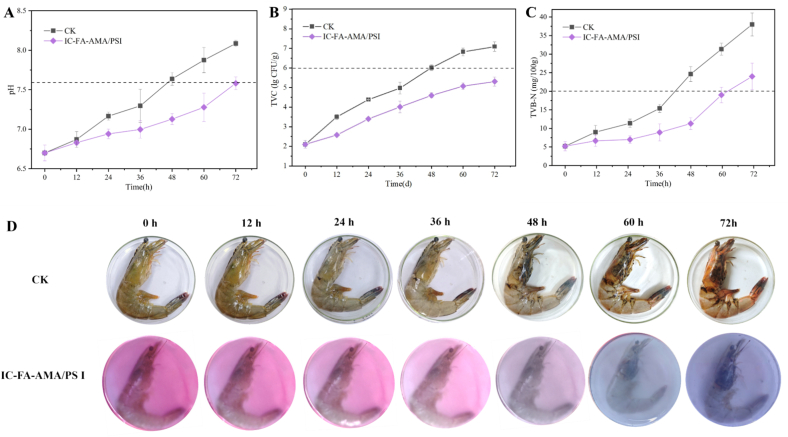
. The pH (A), TVC (B) and TVB-N (C) of the shrimp for different storage at 4 °C times. and ABTS scavenging activity (D). (IC-FA-AMA/PS I: Ferulic acid-*Aronia melanocarpa* anthocyanins/potato starch film with 20 mg/mL IC.)

As shown in Fig. 6E, CL was released most rapidly within 72 h in aqueous food systems, whereas slower release occurred in emulsion-alcohol systems, and the slowest release was observed in lipid-rich foods, reaching equilibrium at 96 h. Although the release rate varied in different food environments, the overall trend was consistent, with blast growth in the early stages and a gradual decrease in release rate as the differences between components decrease, eventually reached equilibrium after complete release (Wu et al., 2023). It could be seen that release behavior of CL from films under different simulation conditions are all Ritger-Peppa models (R^2^ = 0.971, 0.967, 0.970, respectively) from the correlation coefficients R^2^ in Fig. 7B, where the release of CL in aqueous foods, oil-in-water emulsions and alcoholic foods was mainly controlled by Fick diffusion (*n* < 0.45). And the release behavior in the simulated fatty food environment included a combination of distribution and solubilization (*n* = 0.498). Based on the difference in hydrophilicity between CO (alcohol soluble) and PS (water soluble), the release process seems more complex. It may be related to the solubility of the film, the physicochemical properties of the essential oil, and the polarity of the solvent (Lian et al., 2019). In water-based matrices, the hydrophilic nature and water solubility of the PS film expedite the release of CL by disrupting the hydrogen bonds in the film (Li et al., 2023).

Thus, the higher the water content in the food simulant, the faster the release rate of the active intelligent film. This not only effectively extends the shelf life of food but also further enhances the practical application value of the bifunctional film.

### 3.4 Application evaluation to shrimp

During storage, the activation of endogenous proteolytic enzymes in shrimp muscle triggers autolysis, leading to tissue breakdown. This biochemical reaction generates alkaline compounds, resulting in an increase in pH. The pH threshold for shrimp spoilage is typically around 7.6. As shown in Fig. 7A, the pH of the CK group surpassed this limit after 48 h of storage. On the other hand, shrimp preserved with the active intelligent film maintained a pH of 7.58 ± 0.11 even after 72 h, and this stability can be attributed to the gradual release of CL from the film, which effectively suppresses the enzymatic activity in the shrimp and delays the production of alkaline substances (Li et al., 2022).

TVC serves as a standard measure for assessing food spoilage, with values above 6 lgCFU/g indicating unacceptable quality. As shown in Fig. 7B, after 48 h of storage, the TVC for the CK group was 6.02 ± 0.06 lgCFU/g. In contrast, shrimp packaged with the active intelligent film exhibited TVC levels below 6 lgCFU/g even after 72 h. This preservation effect is likely attributed to the inhibition of bacterial growth and reproduction by CL, which is gradually released from the film during storage (Zhang et al., 2023). Laroque et al. (2021) also achieved excellent antibacterial activity by incorporating CL into the cellulose acetate film.

According to the Chinese standard GB 5009.228–2016 (China, N. H. C. o. t. P. s. R. o. C. S. A. o. t. P. s. R. o, 2016b)., the TVB-N level in freshwater fish and shrimp should remain under 20 mg/100 g. For the CK group shrimp, the TVB-N concentration rose markedly from 5.2 to 24.64 mg/100 g after 48 h (Fig. 7C), indicating spoilage. In contrast, shrimp packaged with the active intelligent film showed TVB-N values close to 20 mg/100 g only after 60 h of storage. This delay is linked to the continuous release of CL from the film, which inhibited microbial proliferation and reduced the production of alkaline nitrogen compounds.

Fig. 7D illustrates the color changes of the active intelligent film during shrimp storage. At 4 °C, spoilage of the CK group shrimp was observed, with visible signs of degradation appearing after 48 h. This trend aligned with the observed pH, TVB-N, and TVC values during the same storage period. In comparison, the IC-FA-AMA/PS I film initially displayed a pink color. Over time, the pink hue faded and transitioned to blue by 60 h, with the blue intensity continuing to increase as storage time extended. This shift in color reflects the accumulation of volatile basic substances in the shrimp, which triggers corresponding changes in the color of the film.

Taken together, the findings indicate that the active intelligent film effectively monitors shrimp freshness and increases shelf life by roughly 12 h relative to the control.

## 4 Conclusions

A dual-function intelligent film with high preservation indicator sensitivity and prolonged bacteriostatic effect was constructed in this study. Hydrogen bonds between FA and AMA enhance both the color stability and performance of AMA, thereby improving the reversible color response and storage stability of the film. This enables the film to be used in the future not only for meat and seafood but also for monitoring spoilage of fruits and vegetables. CL was effectively encapsulated by HP-β-CD through hydrogen bonding and hydrophobic forces, leading to a delayed release of CL for approximately 96 h. Such dynamic, long-term bacteriostatic ability could reduce food quality loss and effectively prolong food freshness. This research addresses the current limitations of intelligent films and makes possible their widespread use in future markets.

However, according to the results of physical property tests, further improvements in the transparency and mechanical strength of the film are required. Furthermore, the packaging does not yet offer precise control over the release of active substances. Therefore, the next step in our research is to develop packaging with both controlled-release and indication capabilities for on-demand delivery of active agents.

## CRediT authorship contribution statement

**Liu Yang:** Writing – review & editing, Validation, Supervision, Investigation, Conceptualization. **Xue Ding:** Writing – original draft, Investigation, Formal analysis. **Rui Xiao:** Software. **Xinyu Zhang:** Investigation. **Zhipeng Jiang:** Investigation. **Wenwen Chen:** Investigation. **Shengyu Xu:** Investigation. **Hongyuan Zhang:** Writing – review & editing, Validation, Project administration, Funding acquisition.

## Declaration of competing interest

The authors declare that they have no known competing financial interests or personal relationships that could have appeared to influence the work reported in this paper.

## Data Availability

No data was used for the research described in the article.

## Appendix A Supplementary data

Supplementary material: Color and *L**, *a**, *b**, *ΔE* Value Changes of AMA/PS, FA-AMA/PS, IC-AMA/PS, IC-FA-AMA/PS Ⅰ, and IC-FA-AMA/PS Ⅱ Films During 30-Day Storage at 4 °C and 25 °C. Image 1
