# Supplementary material for: Construction and application of sustained-release active intelligent packaging incorporating phenolic acid copigmented Aronia melanocarpa anthocyanins and microencapsulated carvacrol
Source: Food Chem X. 2025 Dec 13;33:103384. doi: 10.1016/j.fochx.2025.103384 (PMC12769856; doi:10.1016/j.fochx.2025.103384)
Supplement: Supplementary file 1 — Supplementary material: Color and L*, a*, b*, ΔE Value Changes of AMA/PS, FA-AMA/PS, IC-AMA/PS, IC-FA-AMA/PS Ⅰ, and IC-FA-AMA/PS Ⅱ Films During 30-Day Storage at 4 °C and 25 °C. [file mmc1.docx]

**Fig. S1** Color and *L**, *a**, *b**, Δ*E* values of films at 25°C, 4°C. (AMA/PS: *Aronia melanocarpa* anthocyanins/potato starch film; FA-AMA/PS: *Aronia melanocarpa* anthocyanins /potato starch film with ferulic acid; IC-AMA/PS: *Aronia melanocarpa* anthocyanins/potato starch film with 20mg/mL IC; IC-FA-AMA/PS Ⅰ: Ferulic acid-*Aronia melanocarpa* anthocyanins/potato starch film with 20mg/mL IC; IC-FA-AMA/PS Ⅱ: Ferulic acid-*Aronia melanocarpa* anthocyanins/potato starch film with 40mg/mL IC.)
